# Species Richness of Papilionidae Butterflies (Lepidoptera: Papilionoidea) in the Hengduan Mountains and Its Future Shifts under Climate Change

**DOI:** 10.3390/insects14030259

**Published:** 2023-03-06

**Authors:** Xin-Tong Yu, Fei-Ling Yang, Wa Da, Yu-Chun Li, Hong-Mei Xi, Adam M. Cotton, Hui-Hong Zhang, Kuang Duan, Zhen-Bang Xu, Zhi-Xian Gong, Wen-Ling Wang, Shao-Ji Hu

**Affiliations:** 1 Institute of International Rivers and Eco-Security, Yunnan University, Kunming 650500, China; 2 Yunnan Key Laboratory of International Rivers and Transboundary Eco-Security, Yunnan University, Kunming 650500, China; 3 Asian International River Center, Kunming 650500, China; 4 Tibet Plateau Institute of Biology, Lhasa 850008, China; 5 Yulong Xueshan Provincial Nature Reserve, Yulong, Lijiang 674100, China; 6 86/2 Moo 5, Tambon Nong Kwai, Hang Dong, Chiang Mai 50230, Thailand; 7 School of Agriculture, Yunnan University, Kunming 650500, China

**Keywords:** Papilioninae, Parnassiinae, species distribution model, suitability, conservation

## Abstract

**Simple Summary:**

To form a better understanding and benefit future conservation of the apollo and swallowtail butterflies in the Hengduan Mountains in China, the present research projected the 59 Papilionidae species’ spatial richness using the Maxent model and predicted the response to climate change. The spatial pattern of apollos concentrated in the subalpine to alpine areas in western Sichuan, northwestern Yunnan, and eastern Tibet, while that of swallowtails is more confined to the tropical and subtropical valleys of western Yunnan and western Sichuan. Both subfamilies would exhibit northward and upward range shifts with climate change but many apollos would experience drastic habitat contraction, resulting in lower species richness, while most swallowtails would experience habitat expansion, and the species richness would increase. Species with habitat contraction, narrow-ranged distribution and endemicity require more conservation measures in the future.

**Abstract:**

The family of Papilionidae (Lepidoptera: Papilionoidea) is a group of butterflies with high ecological and conservation value. The Hengduan Mountains (HMDs) in Southwest China is an important diversity centre for these butterflies. However, the spatial distribution pattern and the climate vulnerability of Papilionidae butterflies in the HDMs remain unknown to date. The lack of such knowledge has already become an obstacle in formulating effective butterfly conservation strategies. The present research compiled a 59-species dataset with 1938 occurrence points. The Maxent model was applied to analyse the spatial pattern of species richness in subfamilies Parnassiinae and Papilioninae, as well as to predict the response under the influence of climate change. The spatial pattern of both subfamilies in the HDMs has obvious elevation prevalence, with Parnassiinae concentrated in the subalpine to alpine areas (2500–5500 m) in western Sichuan, northwestern Yunnan and eastern Tibet, while Papilioninae is concentrated in the low- to medium-elevation areas (1500–3500 m) in the river valleys of western Yunnan and western Sichuan. Under the influence of climate change, both subfamilies would exhibit northward and upward range shifts. The majority of Parnassiinae species would experience drastic habitat contraction, resulting in lower species richness across the HDMs. In contrast, most Papilioninae species would experience habitat expansion, and the species richness would also increase significantly. The findings of this research should provide new insights and a clue for butterfly diversity and climatic vulnerability in southwestern China. Future conservation efforts should be focused on species with habitat contraction, narrow-ranged distribution and endemicity with both in situ and ex situ measures, especially in protected areas. Commercialised collecting targeting these species must also be regulated by future legislation.

## 1. Introduction

Butterflies are one of the most well-known group of insects with diverse ecological values, including pollinating, ornamental, cultural and scientific values [1]. The Papilionidae (Lepidoptera: Papilionoidea) family, or the commonly known apollos and swallowtails, bears the most significant ecological value among all butterflies, as well as being the subject of the highest conservation interest worldwide [2,3,4]. The Papilionidae family consists of over 600 species in three subfamilies, namely Baroniinae, Parnassiinae and Papilioninae [5]. Except for Baroniinae, which comprises a single species endemic to Mexican deserts, Parnassiinae and Papilioninae species can be found in all non-polar continents except South America [5], where only Papilioninae are found.

China is a great mega biodiversity hotspot in Asia [6,7], with over 2077 species of butterflies recorded to date, while the Hengduan Mountains (HDMs) in Southwest China harbours nearly 65% of the country’s Papilionidae species [8]. Despite this high species richness, research on these butterflies in this particular area has long been limited to inventory surveys and taxonomic studies [9,10,11]; the spatial pattern of these butterflies and its underlying causes as well as responses to climate change remains poorly understood. However, butterflies are sensitive to climate change; temperature and rainfall in particular could affect butterflies’ genetic expression, flight-related morphological characters, life history and voltinism, trophic relationships, seasonality and phenology, behaviour and richness distribution pattern [12,13,14,15,16]. Highly mobile species could even achieve intercontinental expansion rapidly [17]. Therefore, the lack of such knowledge has already become an obstacle in forming effective butterfly conservation strategies for this area [1].

In recent years, with intensifying anthropogenic disturbances, including climate change, habitats in the HDMs have undergone obvious degradation [18]. Butterflies are very sensitive to habitat degradation and would suffer from various negative consequences ranging from population decline to fragmentation, as stated in a recent typical report for *Bhutanitis thaidina* [19,20,21], a National Grade II Protected butterfly species in China [4]. A similar study on a cold-adapted butterfly in Europe further demonstrated that, behind this distribution change, genetic diversity is also at risk when locally unique haplotypes are less suitable [22]. Studies on migratory species such as *Danaus plexippus* (Lepidoptera: Nymphalidae) showed that climate change could influence the butterfly’s suitable habitats directly via ambient temperature, precipitation and indirectly via the potential distribution range of its larval food plants [23,24]. To protect the Papilionidae diversity and its ecological values in the HDMs, holistic conservation research is ultimately necessary, and the spatial pattern of species richness and responses to climate change is essential [25].

The present research aimed at this essential question, and thus analysed the current spatial pattern and the future shift under different future climatic scenarios [26] using Maxent modelling and further spatial analysis, the results of which will deepen our understanding of the spatial diversity character and its underlying effects on Papilionidae butterflies in the HDMs, as well as provide an insight into how the diversity pattern would change in the process of climate change. The findings will benefit future conservation management and the formulation of a more effective conservation strategy for the butterflies in this particular area.

## 2. Materials and Methods

### 2.1. Research Area

**Figure 1:**
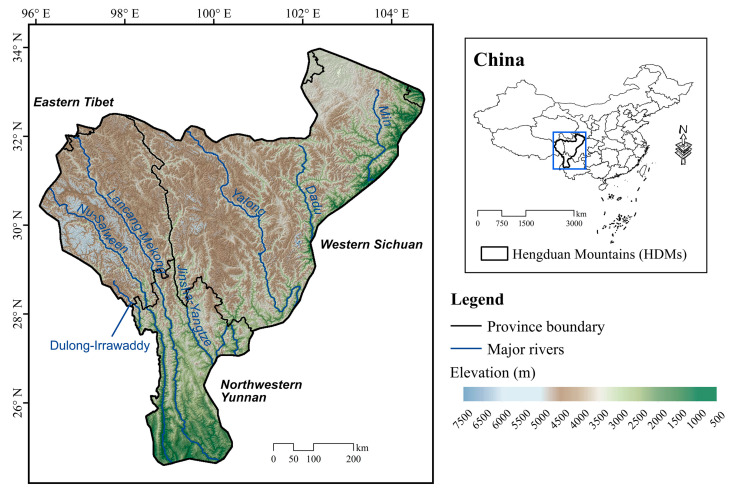
The location and topography of the Hengduan Mountains (HDMs).

Our research area covers the entire HDMs of Southwest China (approximately 95° to 105° E and 24° to 34° N), which mainly consists of eastern Tibet, western Sichuan and northwestern Yunnan, with an area of about 490,000 km^2^ (Figure 1, Figures S1 and S2). The boundary of the HDMs was drawn by compiling multiple descriptions from two publications [27,28].

The topography of the HDMs is represented by tremendous elevational relief within a limited range; the vast western and northern parts of the HDMs are situated in the Qinghai-Tibet Plateau, while the eastern and southern margins are lower elevation areas and between the two extremes is a steep transition zone [28]. Four river systems, namely Dulong-Irrawaddy River, Nu-Salween River, Lancang-Mekong River, and Jinsha-Yangtze River, are well developed, with deep-cutting valleys from the west to the east [29].

### 2.2. Species Records

Species distribution points were extracted from (1) specimen records in museums and academic institutions, (2) specimen records in private collections of the authors, (3) public biodiversity databases and (4) literature records. Museums and academic institutions include: the Natural History Museum (London, UK), Institute of Zoology, Chinese Academy of Sciences (CAS) (Beijing, China), Kunming Institute of Zoology, CAS (Kunming, China), Southwest Forestry University (Kunming, China), Yunnan University (Kunming, China), Dali University (Dali, China) and Wenshan College (Wenshan, China). Public biodiversity databases are: Global Species Diversity Information Database (GBIF, www.gbif.org (accessed on 1 March 2020)) and China National Specimen Resource Platform (NSII, www.nsii.org.cn (accessed on 1 March 2020)). Literature records include those published in taxonomic papers and manuals [8,30,31,32,33,34,35,36,37,38,39], biodiversity surveys [40,41] and regional checklists for butterflies [42,43].

Records with precise sites or with coordinates were chosen, and all obtained or translated coordinates were stored in decimal degrees in an Excel spreadsheet for further use. All species records were double checked in Google Earth to ensure accuracy, and then loaded in ArcMap 10.2 to eliminate duplicated and spatially over-close records (distance < 5 km) to minimise the bias caused via spatial autocorrelation [44]. In total, the present research obtained 59 Papilionidae species with 1938 presence data points, with the number of data points of *Byasa polyeuctes* being the most (101) and that of *Papilio macilentus* being the least (7) (Table S1). A total of 84.2% (1632) of the used data points were recorded within the time period between 1970–2000.

### 2.3. Environmental Variables

Nineteen BioClim variables (Table S2) were used to represent the current climate features (averaged over the period ranging from 1970–2000). Climate System Model version 1.1 (BCC-CSM1.1) developed by the Beijing Climate Centre was adopted for future climates as a good representation in Asia [45]. For future climates, two future periods, namely 2050s (average for between 2041–2060) and 2070s (average for between 2061–2080), with three carbon emission scenarios (RCP2.6, RCP4.5 and RCP8.5) [26], were each used in subsequent analyses. All climate data were downloaded from the WorldClim database (www.worldclim.org (accessed on 19 October 2019)) [46,47].

All environmental variables were downloaded in 30 arc seconds resolution (≈1 km) and transformed to the GCS-WGS-1984 coordinate system in ArcGIS 10.4 (ESRI, Redlands, CA, USA). The resultant data were further cropped by the boundary of the HDMs and output as ASCII format.

To minimise the interference of auto-correlated BioClim variables, the 19 variables were screened using the Pearson correlation method using the ‘corrplot’ package [48] in R 3.6.1 (https://www.r-project.org/ (accessed on 16 July 2019)). For variables with |*r*| > 0.75, the short period extremes, e.g., temperature of the driest/wettest month, precipitation of the coldest/warmest month, were excluded from the dataset using a stepwise strategy until the remaining variables’ |*r*| < 0.75 [19]. Using this criterion, variables Bio2, Bio5, Bio6, Bio8, Bio9, Bio13, Bio14, Bio18 and Bio19 were excluded from the subsequent analyses (Figure S3).

### 2.4. Maxent Simulation and Spatial Richness Analysis

Maxent 3.4.1 was used to project the potential distribution of the Papilionidae species in the HDMs under current and future climates using the presence data points [49]. For each species, 75% of the total points were selected for training, while the remaining 25% were kept for testing, and 10 replications were run to achieve the best simulation. Model robustness for each species was evaluated using the receiver operation curve (ROC) and the area under the ROC curve (AUC, Table S1), where the AUC value [AUC∈(0, 1)] approaching 1 indicates acceptable, whereas it should be rejected when approaching the random prediction of 0.5 [50]. To determine the stability of the Maxent simulation under all climate scenarios, an ANOVA analysis using the AUC values of all species was performed in SPSS 28 (IBM Inc., Armonk, NY, USA) to test statistical differences. The key influencing BioClim variables for each species under all climate scenarios were determined using the jackknife method in Maxent.

The output of the Maxent analysis is the probability of distribution, which ranges from 0 to 1, where the probability approaching 1 indicates a higher likelihood of actual distribution. To interpret prediction results into binary classifications (presence—1 or absence—0), a threshold must be adopted. When prediction values are greater than the threshold, the binary value ’1‘ is used, while the value ’0‘ is assigned when the prediction is less than the threshold. To obtain a better accuracy and minimise false interpretation, the present research did not use 0.5 as the threshold but employed the concept of categorising uncertainties stated in the IPCC Reports on Climate Change and adopted 0.66 (rank ’likely‘) as the threshold for distribution (presence) [51,52]. Then, for each Maxent resultant grid file, the grid cells with probability values ≥ 0.66 were interpreted into a binary value of ’1‘ and extracted to form a new grid file to represent the distribution area of a species.

The distribution maps of different Papilionidae species were then stacked in ArcGIS 10.4 to obtain the species richness maps under current and future climates. Since the biological and ecological characters of Parnassiinae and Papilioninae species are very different (see Introduction), maps of the two subfamilies were produced separately. Species richness ranks were calculated and divided based on the number of species (*S*) in a single grid cell. To compare species richness under current and future climates, changes in species richness (Δ*S*) in a single grid cell were also calculated. Based on the species presence in a grid cell, the occupancy areas of each species under current and future climates were also calculated and compared.

## 3. Results

### 3.1. Model Performance and Key BioClim Variables

Under current climate conditions, the AUC values range from 0.783–0.992 (mean = 0.954 ± 0.053), with that of *Parnassius imperator* being the smallest, while that of *Graphium tamerlanus*, *Byasa latreillei* and *Meandrusa sciron* being the greatest together. Over 86% of species’ AUC values were over 0.9, while only 5.1% were below 0.8 (all in Parnassiinae) (Table S1). Under future climate scenarios, the smallest AUC values ranged from 0.764–0.807, and the greatest values ranged from 0.992–0.996. The percentage of AUC values above 0.9 ranged from 84.7–88.1%, and that below 0.8 ranged from 0–5.1%. Under the three future climate scenarios in the 2050s, *M. sciron* gained the greatest AUC values and *P. imperator* gained the smallest AUC values, while in the 2070s, *G. tamerlanus* gained the greatest values and *P. epaphus* gained the smallest AUC values (Table S1). Statistical comparisons showed no significant differences between all climate scenarios (ANOVA *p* > 0.05), demonstrating a stable Maxent performance under all used climate scenarios.

Among the 19 BioClim variables, Bio10 (mean temperature of warmest quarter) is the most influencing one, followed by Bio12 (annual precipitation), Bio1 (annual mean temperature) and Bio11 (mean temperature of coldest quarter). In the remaining six variables, Bio15 (coefficient of variation of precipitation seasonality) and Bio16 (precipitation of wettest quarter) are far less important compared to Bio3 (isothermality), Bio7 (temperature annual range), Bio17 (precipitation of driest quarter) and Bio4 (temperature seasonality).

### 3.2. Current Distribution Pattern

**Figure 2:**
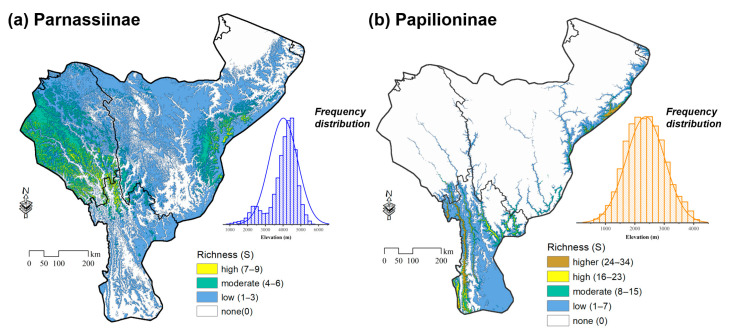
Spatial distribution pattern of species richness for Parnassiinae (**a**) and Papilioninae (**b**) in the HDMs projected under current climate condition. The frequency distribution of suitable elevation ranges for Parnassiinae (blue) and Papilioninae (orange) species in the HDMs.

Under current climate conditions, the suitable areas of the 14 Parnassiinae species range from 8984–111,058 km^2^, with the range of *Parnassius epaphus* being the broadest, while that of *P. labeyriei* is the narrowest (Table S3). The highest species richness of Parnassiinae in the HDMs reaches nine species, occupying multiple high-elevation mountains in eastern Tibet (Figure 2a). Areas with high species richness ranges from 4 to 7 are found neighbouring to the aforementioned area, as well as in northwestern Yunnan and near Kangding of western Sichuan. The remaining vast area in the HDMs possesses relatively lower species richness (1–4), covering 49.24% of the entire research area. Our analyses also yield non-suitable areas (36.70%) in the far northeast corner of the HDMs along with all river valleys below 1500 m.

For the 45 Papilioninae species, the suitable areas range from 1171–64,906 km^2^, with the range of *Graphium confucius* being the broadest, while that of *Byasa latreillei* is the narrowest (Table S3). The highest richness, 34 species, is found in the valleys of the Dulong-Irrawaddy River valley in northwestern Yunnan (Figure 2b). High species richness (24–34) is also found in the valley of the Nu-Salween River in western Yunnan and near Dujiangyan located in the conjunction area of the HDMs and the Sichuan Basin; and the medium–high species richness (8–23) is identified in parts of the four river valleys, especially the Jinsha-Yangtze River and the upper Lancang-Mekong River. The lower richness (1–7) areas are mostly the medium-elevation mountain slopes and the upper section of all river valleys in eastern Tibet and western Sichuan, covering 13.90% of the research area. The remaining higher elevation areas, including the vast areas in western Sichuan and eastern Tibet as well as the high mountains in northwestern Yunnan, are not suitable for Papilioninae species.

It is obviously notable that the suitable distribution ranges of Parnassiinae and Papilioninae species are largely different spatially, despite small overlapping areas in western Yunnan and western Sichuan (Figure 2). Our statistics shows that Parnassiinae species mainly occupied the mountainous regions with elevation ranges from 2500 to 5500 m, while the Papilioninae species preferred lower elevations from 1500 to 3500 m (Figure 2).

### 3.3. Future Changes

#### 3.3.1. Parnassiinae

**Figure 3:**
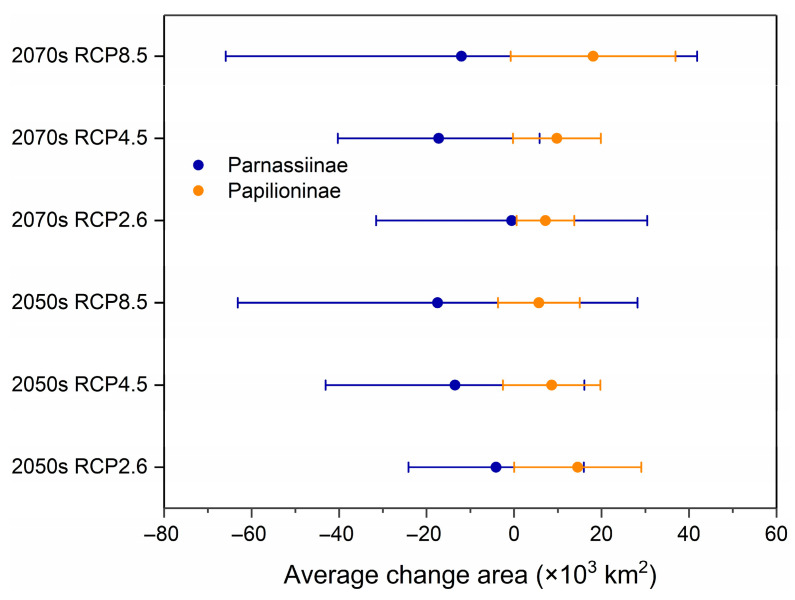
Average change in Parnassiinae and Papilioninae potential distribution areas under different climate scenarios; circles are average change areas, bars represent standard errors.

The results of the average change area of the species potential distribution indicated that with future climate warming, the distribution area of Parnassiinae in the HDMs will generally decrease, and this trend will be more obvious as the climate change scenario changes from mild to extreme (Figure 3). Specifically, for *Parnassius* species, the general trend obtained via our analysis is a reduction in suitable areas, except for *Parnassius imperator* and *P. orleans*, whose suitable areas would increase under all future climate scenarios (Table S3). For *Bhutanitis* species, *Bhutanitis mansfieldi* and *B. thaidina* would gain more suitable areas under RCP2.6 and RCP4.5 scenarios, while *B. lidderdalii* would lose its suitability under most future climate scenarios (Table S3).

**Figure 4:**
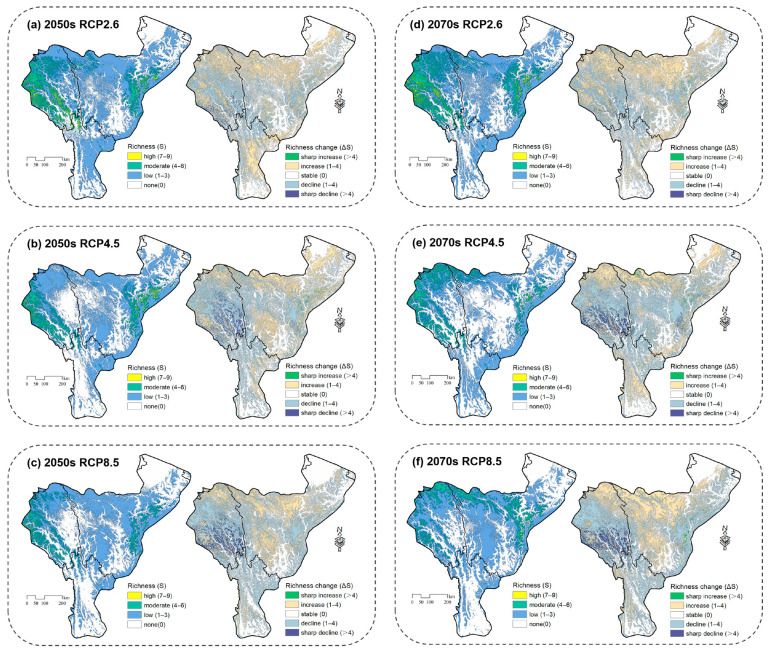
Species richness and changes in Parnassiinae in HDMs under current and different climate scenarios. Subfigures represent richness (**left**) and changes (**right**) under: (**a**) future climate scenario RCP2.6 in 2050s, (**b**) future climate scenario RCP4.5 in 2050s, (**c**) future climate scenario RCP8.5 in 2050s, (**d**) future climate scenario RCP2.6 in 2070s, (**e**) future climate scenario RCP4.5 in 2070s, and (**f**) future climate scenario RCP8.5 in 2070s.

**Figure 5:**
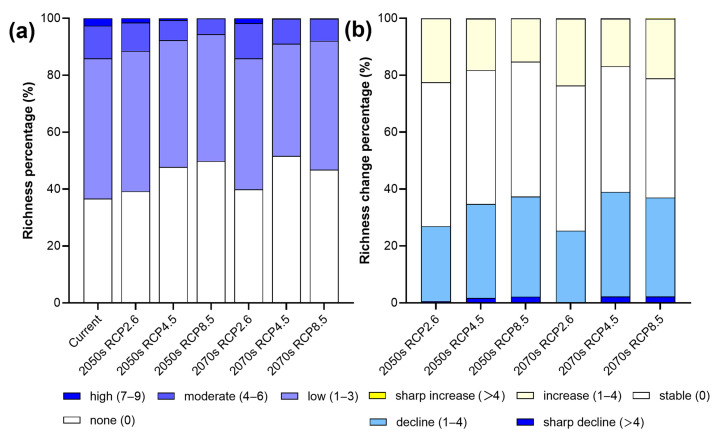
Percentage of species richness (**a**) and changes (**b**) (by the number of grid cells) in Parnassiinae under different climate scenarios.

In the 2050s, under the RCP8.5 scenario, the richness of Parnassiinae would decrease by 37.40%, which is more than double the increase (15.23%) in some areas (Figure 4), implying that the richness of Parnassiinae would reduce notably. Meanwhile, areas with high richness would gradually shift to medium richness. Compared with the current status, the principal distribution of Parnassiinae would shift to the high-elevation areas, such as Basu and Karuo, followed by Kangding, Luding and Xiaojin (Figure 5). However, under the RCP2.6 scenario, the change in each richness level is less than 2.51% (Figure 5). In terms of spatial pattern, the species richness would decrease in the central mountains and the southern margin of the study area, whereas the richness would rise in the northern high-elevation areas. Under the RCP4.5 scenario, the non-suitable areas would increase by more than 10%, while the change in other richness ranks is less than 4.60% compared to the current status (Figure 5). The overall spatial pattern of species richness is similar to that under the RCP2.6 scenario, but the degree of shifting is more obvious.

In the 2070s, the richness of Parnassiinae would decline under the three scenarios. Overall, the species richness would decrease at low elevations and increase at the higher elevations. In detail, the shift in distribution to higher elevations would become more obvious under extreme climate scenarios. Under the RCP8.5 scenario, areas with high richness would be very limited, and the distribution would shift to extremely high elevations near the northern boundary of the study area, such as Jiangda and Dege (Figure 4). Compared to the 2050s, especially under the RCP4.5 scenario, the non-suitable areas comprise more than half of the study area (Figure 5), and the reduction in species richness is more severe. In contrast, under the RCP2.6 and RCP4.5 scenarios, the decline in richness would be moderate.

#### 3.3.2. Papilioninae

Our analysis discovered that the majority of Papilioninae would expand their distribution under the influence of climate change. By the 2050s, under the RCP2.6 scenario, the distribution of Papilioninae would change most significantly, with an average change of 15,300 km^2^ (Figure 3). Among the 45 species, over 90% of species would expand their distribution range, with that of *Pachliopta aristolochiae* being the greatest (Table S3). By the 2070s, under the RCP8.5 scenario, the average expansion of distribution would reach 19,001 km^2^, with over 80% of species expanding their ranges, that of *Teinopalpus imperialis* being the greatest.

**Figure 6:**
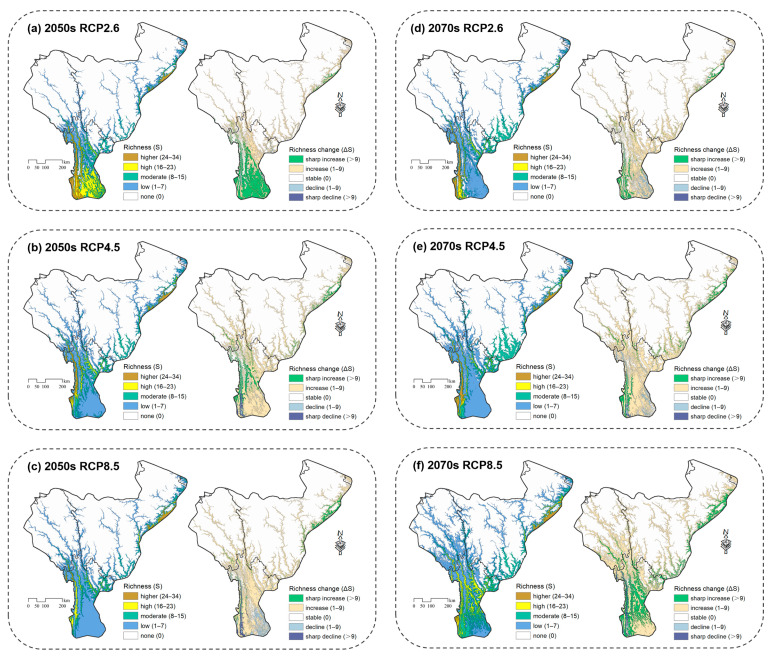
Species richness and changes in Papilioninae in HDMs under current and different climate scenarios. Subfigures represent richness (**left**) and changes (**right**) under: (**a**) future climate scenario RCP2.6 in 2050s, (**b**) future climate scenario RCP4.5 in 2050s, (**c**) future climate scenario RCP8.5 in 2050s, (**d**) future climate scenario RCP2.6 in 2070s, (**e**) future climate scenario RCP4.5 in 2070s, and (**f**) future climate scenario RCP8.5 in 2070s.

**Figure 7:**
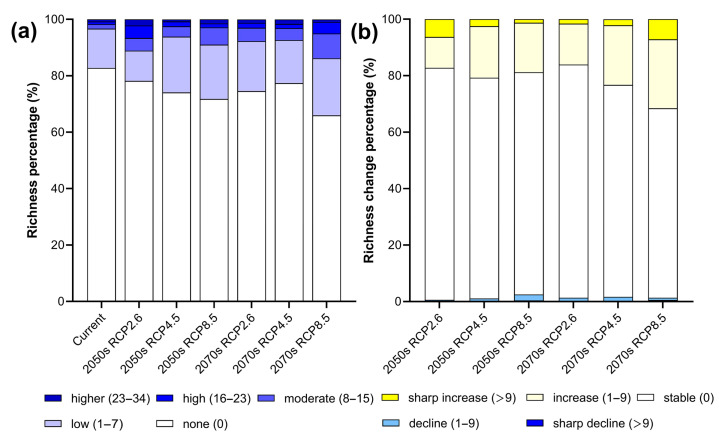
Percentage of species richness (**a**) and changes (**b**) (by the number of grid cells) in Papilioninae under different climate scenarios.

In the 2050s, the non-suitable area for species richness would decrease under all three scenarios (Figure 6). Regionally, species richness would increase significantly in the southern part of the study area and expand towards higher elevations. Similar increasing patterns are observed under the RCP2.6 and RCP8.5 scenarios. Accordingly, the richness increase would reach maximum under the RCP4.5 scenario. It is noteworthy that the richness increase at higher elevations is more remarkable under the RCP8.5 scenario. The areas with higher species richness are found near Lushui, Tengchong and Dujiangyan (Figure 7).

In the 2070s, under the three scenarios, species richness would further increase to higher elevations along multiple river valleys (Figure 7). This trend is more typical under the RCP8.5 scenario compared to the current status, with a net increase over 30% in the study area.

## 4. Discussion

### 4.1. Causes Underlying Species Richness Pattern

Our analysis revealed that the spatial distribution pattern of Parnassiinae and Papilioninae species are quite different in the HDMs. A high species richness of Papilioninae is mainly found in the valleys of Dulong-Irrawaddy, Nu-Salween and the Lancang-Mekong rivers, as well as the valleys near Dujiangyan (Figure 2b), where the climate is warmer and humid [53]. Meanwhile, most Parnassiinae species, especially genus *Parnassius*, are found in high elevation habitats on the Tibetan Plateau, where the climate is generally cooler and drier [53] (Figure 2a).

It must be mentioned that the species richness of butterflies is more directly linked to regional vegetation diversity [54], which can serve as a pool of food plants for butterflies. For Parnassiinae species, their food plants are mainly plant species in the families Papaveraceae (sensu lato), Crassulaceae, Scrophulariaceae and Aristolochiaceae [33,55]. Families Papaveraceae (s.l.) and Crassulaceae are common in higher elevation areas, while certain Aristolochiaceae species, e.g., *Aristolochia moupinensis*, could be found in medium–high elevation areas [56]. In particular, Papaveraceae (s.l.) plants underwent drastic divergence and reached very high diversity during the tectonic shifts of the Himalayas and the HDMs [57,58,59,60], while Crassulaceae plants prefer arid habitats and are commonly found in alpine screes. Most species of the two families are very abundant in the HDMs, as confirmed by our field surveys in this research. For Papilioninae species, their food plants are more diverse across several plant families, e.g., Magnoliaceae, Lauraceae, Annonaceae, Hernandiaceae, Rosaceae, Aristolochiaceae and Rutaceae [61]. The food plants in these families are mainly found in subtropical and tropical areas in the HDMs, with a higher diversity in river valleys [62,63,64]. The concentration of these plants as well as the associated butterflies might be attributed to the climate oscillations since the Last Glacial Maximal, especially in the Pleistocene [65,66,67,68,69].

**Figure 8:**
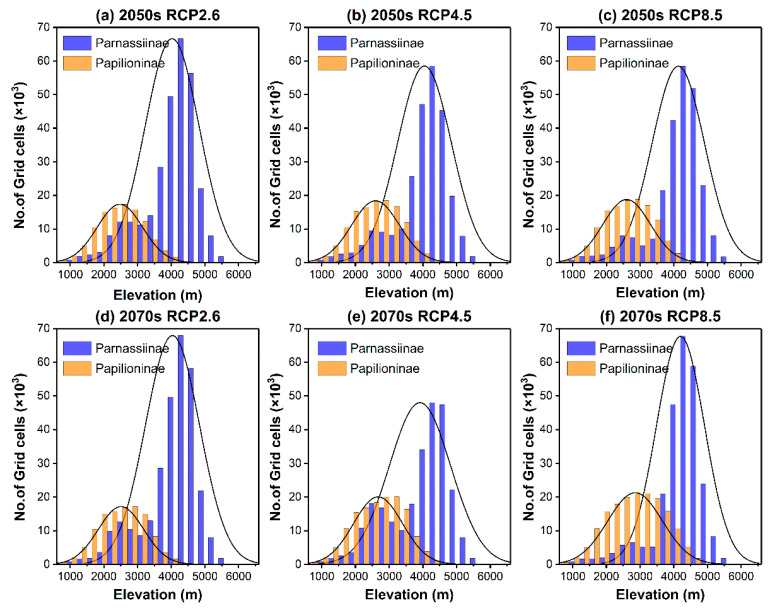
The frequency distribution of suitable elevation ranges for Parnassiinae (blue) and Papilioninae (orange) species in the HDMs under different times. Black curve in each plot is model–fit for frequency distribution. Subfigures represent frequency distributions under: (**a**) future climate scenario RCP2.6 in 2050s, (**b**) future climate scenario RCP4.5 in 2050s, (**c**) future climate scenario RCP8.5 in 2050s, (**d**) future climate scenario RCP2.6 in 2070s, (**e**) future climate scenario RCP4.5 in 2070s, and (**f**) future climate scenario RCP8.5 in 2070s.

It is noteworthy that our analysis produced hump-shaped distribution patterns along the elevation gradients for both Parnassiinae and Papilioninae in the HDMs, even under different future climate scenarios (Figure 2 and Figure 8). Similar studies in the Guadarrama Mountains of Spain [70] and at Mount Hermon in Israel [71] also showed hump-shaped distribution patterns of butterfly richness against elevations. To date, two richness patterns along elevation gradients have been identified, with the first being the decreasing pattern with elevations and the second being the hump-shaped pattern in which species richness peaks near the intermediate elevation sections [72]. The relationship between species richness and elevation has not been fully understood, but generally depends on the focal taxa and gradients considered [72]. Nogues-Bravo et al. [73] argued that, when the entire elevation gradients were surveyed, the results are usually hump-shaped; however, such a relationship would likely progressively turn into a decreasing pattern when the scale diminishes. Similar findings were reported on several vascular plant groups [74], fern species [75] and seed plants [76], which may also contribute to the richness pattern of butterflies.

### 4.2. Climate Vulnerability

Under future climate scenarios, a common trend is that most species would experience range shifts to higher latitudes [77] and elevations [78,79], while contraction of suitable habitats is likely during the process. In the present research, Parnassiinae species in the HDMs would typically face habitat contraction in the future. In the case of the extreme climate scenario (RCP8.5), some species (e.g., *Parnassius labeyriei*) could experience drastic habitat contraction towards mountaintops in Basu, Chayu and Zuogong areas (Figure S4). Since all Parnassiinae involved in this research are non-migratory [80], such a contraction trend would likely push some species to the margin of extinction, as, on the one hand, the distribution shift rate of species can hardly keep up with the pace of habitat shift and contraction [81], and, on the other hand, the ascent of the upper part of its suitable range will be limited by unfavourable climate or vegetation conditions [82]. A case study on 16 mountain-restricted butterflies in Spain showed that climate warming has already reduced their suitable habitats by over 30% in the past three decades [83]. Maclean and Wilson [84] carried out a meta-analysis comparing 188 predicted and 130 observed responses to climate warming in the published literature and concluded that 10–14% of species would be at risk of extinction. Nonetheless, a few Parnassiinae species, such as *Parnassius imperator* and *P. orleans*, two species found in wider elevation ranges [33,85], would expand their distribution area, even under future climate scenarios (Figure S4). Future research is required to elucidate the biological, behavioral and ecological mechanism underpinning the range expansion of such species.

In contrast, the majority of Papilioninae species in our research would expand their distribution ranges under climate change, and the extent of expansion would increase with future climate scenarios. According to Chowdhury et al. [80], *Graphium doson*, *G. sarpedon*, *Pachliopta aristolochiae*, *Papilio agestor*, *P. helenus*, *P. machaon*, *P. memnon*, *P. polytes* and *P. xuthus* are migratory species, which showed great extents of range expansion in our analysis (Table S3). Our speculation is that, under the influence of climate change coupled with the complex topography of the HDMs, more suitable habitats would become available to these species, providing that searching for novel habitats is part of their behaviour. For other species, the expansion of food plants with climate change may contribute to their range shifts. Liang et al. [86] stated that plants living in mountain habitats would also expand their ranges when climate change occurs, providing that complex topography may accommodate those plants within a certain elevation range. As a result, under the influence of climate change, the centres of the species richness of Papilioninae species would also expand towards higher latitudes and elevations. Such a northward shift is not only consistent with multiple cases from Europe, America and the Korean Peninsula reported by Taheri et al. [25], but also supported by the results of other phyla represented by plants [87], birds [88] and mammals [89].

### 4.3. Conservation Implications

Protected areas have great potential in conserving butterfly diversities globally; however, the current identification and establishment of protected areas is biased towards flagship vertebrates, and insects in general are often overlooked [90,91]. For a butterfly diversity centre such as the HDMs, taking this biodiversity dimension into consideration under the latest conservation target of China [92,93] is also important to serve the Kunming-Montreal Global Biodiversity Framework (after the Post-2020 Global Biodiversity Framework) [92,94].

It is evident that both Parnassiinae and Papilioninae species possess unequal spatial richness patterns, hence conservation measures must be taken based on systematic conservation planning, especially the identification of priority conservation areas by including butterfly diversity data alongside the currently used vertebrates and plants [1,94,95,96,97,98,99]. For high richness areas lying inside existing protected areas, regular monitoring schemes are needed to ensure our understanding of their population trends in the future, as well as to identify natural and anthropogenic threats in time [100,101,102]. For high richness areas outside protected areas, further evaluations must be performed to verify the ecological value of adding these areas to current conservation networks. For threatened, endemic and narrow-ranged species (e.g., *Graphium sichuanica*, *Byasa rhadinus*), this work would be more urgent than commonly and widely distributed species [103], especially when establishing ex situ refugia or restoration areas becomes the very last option, given that over 75% of insect species are inadequately represented by protected areas globally [104]. Furthermore, future conservation planning must also consider the concept of ’other effective area-based conservation measures (OECMs)’ [105] to establish in situ conservation outside existing protected areas with local communities, coupled with the diverse ethnic groups and their traditional and local ecological knowledge (TEK and LEK) in the HDMs [1,106].

Under climate change, most Parnassiinae species (non-migratory) would experience significant habitat contraction in the future, and the extinction risk would escalate. For those species at risk, it would be beneficial to carry out research and surveys to identify feasible refugia or ex situ restoration areas to host them, especially in areas such as Zuogong and Basu in Tibet, and Kangding in Sichuan Province. When selecting refugia sites, surveys on vegetation and food plants must be carried out in advance to ensure that the optimal vegetation types are included. For Papilioninae species, although most species would expand their distribution ranges, distinguishing migratory and non-migratory species might be important in formulating correct habitat conservation and management strategies [80]. For species requiring a certain extent of disturbances, suitable type and magnitude of disturbance must also be maintained [107]. Regular monitoring in current species richness centres is essential to understand where and when the above-mentioned conservation measures should be implemented.

For certain oligophagous species [61], responses to climate change are often synchronised with the change in their foodplants [54,108]. Filazzola et al. [109] concluded that the range contraction of *Parnassius smintheus* was directly linked to its host plant reduction under climate change. Similar results were also reported for *Danaus plexippus* (Lepidoptera: Nymphalidae) in the Americas [54,110] and *Anthocharis cardamines* (Lepidoptera: Pieridae) in Europe [111]. Despite the possibility that some butterflies might obtain novel ecological opportunities via host shifts during the course of range shifting, like other herbivorous insects [112,113], conserving and restoring areas with indigenous food plants outside the current distribution margins of these species are equally crucial.

Papilionidae butterflies are the group mostly targeted by commercialised collecting worldwide, and species in the HDMs have long been impacted by such activities, both domestically and internationally. For Chinese endemic species, e.g., *Bhutanitis thaidina*, *B. mansfieldi*, *Byasa rhadinus*, *B. daemonius*, as well as many *Parnassius* living on the Tibetan Plateau, stress from commercialised collecting is more severe (S. J. Hu, unpublished data). Due to insufficient population information, assessments of the species’ statuses are difficult. Future surveys are required to encourage more assessments, which will ultimately benefit our legislation of protected species [1].

## 5. Conclusions and Limitation

The spatial pattern of species richness of the subfamilies Parnassiinae and Papilioninae in the HDMs has obvious elevation prevalence. Parnassiinae is concentrated in the subalpine and alpine areas (2500–5500 m) in western Sichuan, eastern Tibet and northwestern Yunnan, while Papilioninae is concentrated in the low- to medium-elevation areas (1500–3500 m), mainly in the valleys of Dulong-Irrawaddy, Nu-Salween and the Lancang-Mekong rivers in Yunnan, as well as the valleys near Dujiangyan in Sichuan.

The species richness of both subfamilies showed a hump-shaped distribution pattern. Complex topography and regional vegetation diversity resulting from historical tectonic shifts and climate oscillation could largely contribute to the species richness. Under the influence of climate change, the overall distribution of Parnassiinae and Papilioninae species would shift to higher elevations. At least eight species of Parnassiinae would experience habitat contraction, resulting in a decrease in species richness across the HDMs. On the contrary, at least 33 species of Papilioninae butterflies would experience habitat expansion, leading to increased species richness in affected areas.

To conserve Papilionidae butterflies in the HDMs, regular monitoring of species in affected areas, especially those endemic and narrow-ranged species, is essential to obtain population trend information. In situ conservation measures and ex situ refugia selection are also vital to mitigate the impact on species survival from the changing climate.

Although comprehensive, the present research only projected the species richness and future change using abiotic environmental factors, therefore the results might contain discrepancies from actual distribution patterns and future changes. When applying the results to guide field surveys and policy making, it is crucial to be cautious that any species’ spatial distribution should be subject to more complex biotic and even anthropogenic factors.

## Data Availability

All data supporting reported results can be found in the Supplementary Materials. Species distribution records containing coordinates and the original suitability maps are not openly available due to data sensitivity.

## Supplementary Materials

The following supporting information can be downloaded at: https://www.mdpi.com/article/10.3390/insects14030259/s1, Table S1. Species records and AUC values of Maxent analysis; Table S2. Environmental factors and their interpretation; Table S3. Suitable area and changes of Papilionidae butterflies in the Hengduan Mountains (HDMs) under different climatic scenarios; Figure S1. Location of the counties in the HDMs mentioned in this research; Figure S2. Land use types in the HDMs; Figure S3. Pearson correlation matrices of the 19 BioClim variables, circle sizes are proportional to the absolute correlation coefficient values, red colour represents minus and blue colour represents plus; Figure S4. The projected distributional maps for the species *Parnassius labeyriei*, *P. imperator* and *P. orleans* in the HDMs under climate change.
