# Supplementary material for: Species Richness of Papilionidae Butterflies (Lepidoptera: Papilionoidea) in the Hengduan Mountains and Its Future Shifts under Climate Change
Source: Insects. 2023 Mar 6;14(3):259. doi: 10.3390/insects14030259 (PMC10058169; doi:10.3390/insects14030259)
Supplement: Supplementary file 1 [file insects-14-00259-s001.zip › Figure S3.pdf]

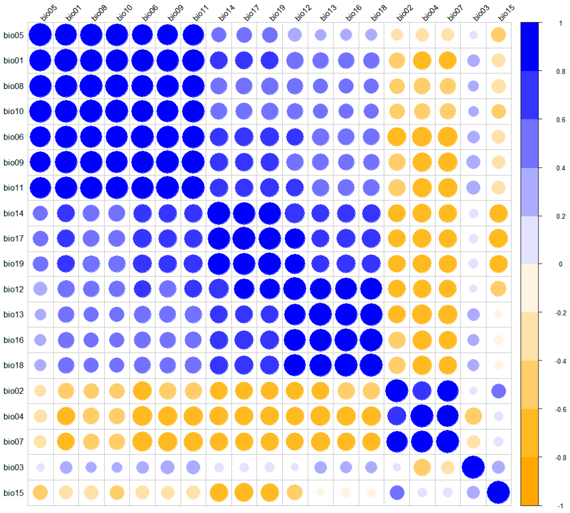

(a) Current

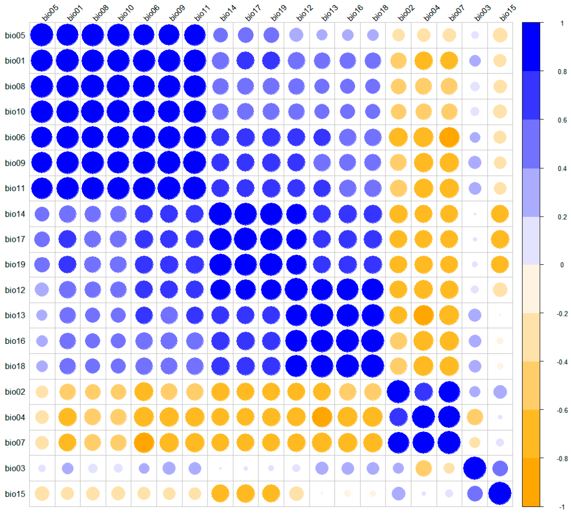

(b) RCP2.6 2050

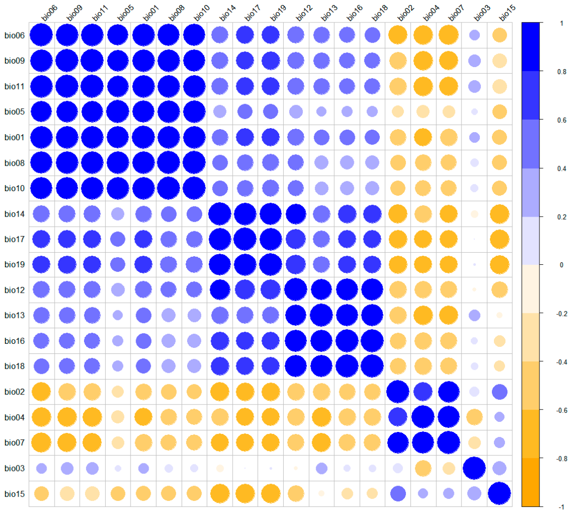

(c) RCP4.5 2050

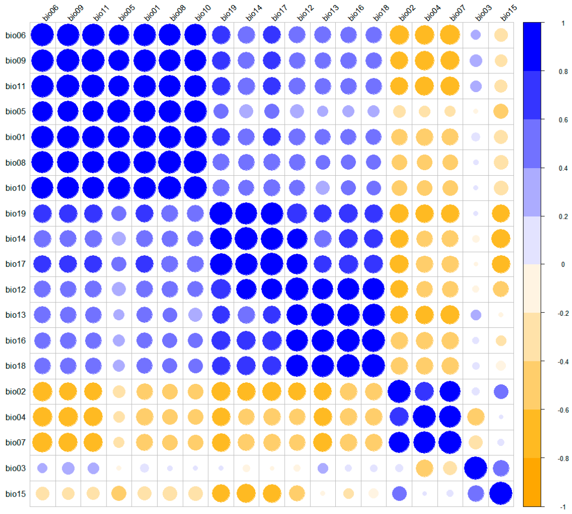

(d) RCP8.5 2050

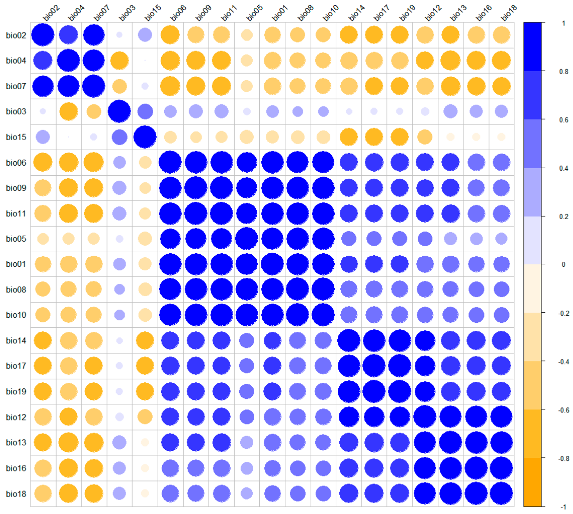

(e) RCP2.6 2070

(*continued*)

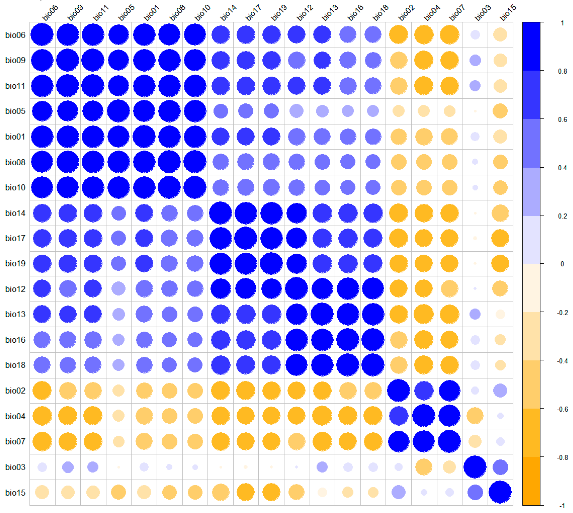

(f) RCP4.5 2070

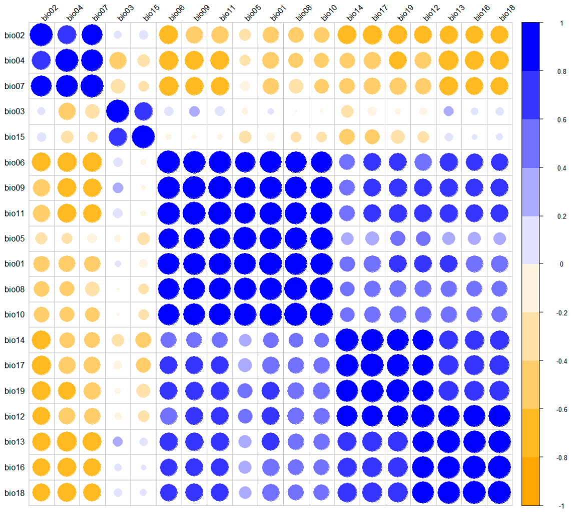

(g) RCP8.5 2070

Figure S3. Pearson correlation matrices of the 19 BioClim variables, circle sizes are proportional to the absolute correlation coefficient values, red colour represents minus and blue colour represents plus.
