# Supplementary material for: Species Richness of Papilionidae Butterflies (Lepidoptera: Papilionoidea) in the Hengduan Mountains and Its Future Shifts under Climate Change
Source: Insects. 2023 Mar 6;14(3):259. doi: 10.3390/insects14030259 (PMC10058169; doi:10.3390/insects14030259)
Supplement: Supplementary file 1 [file insects-14-00259-s001.zip › Figure S4.pdf]

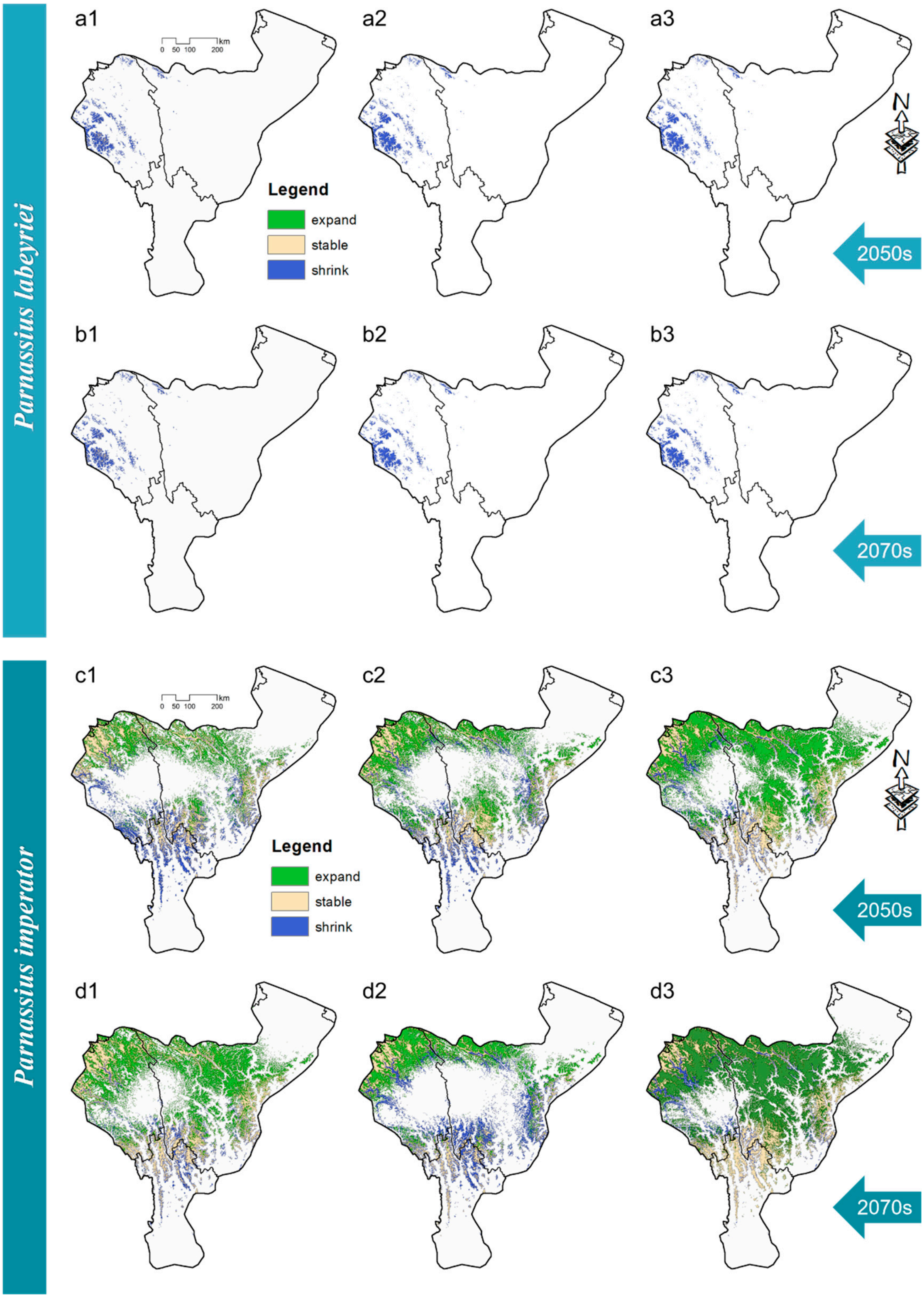

Parnassius labeyriei
a1
a2
a3
0 50 100 200 km
Legend
expand
stable
shrink
N
2050s
b1
b2
b3
2070s
Parnassius imperator
c1
c2
c3
0 50 100 200 km
Legend
expand
stable
shrink
N
2050s
d1
d2
d3
2070s

(*continued*)

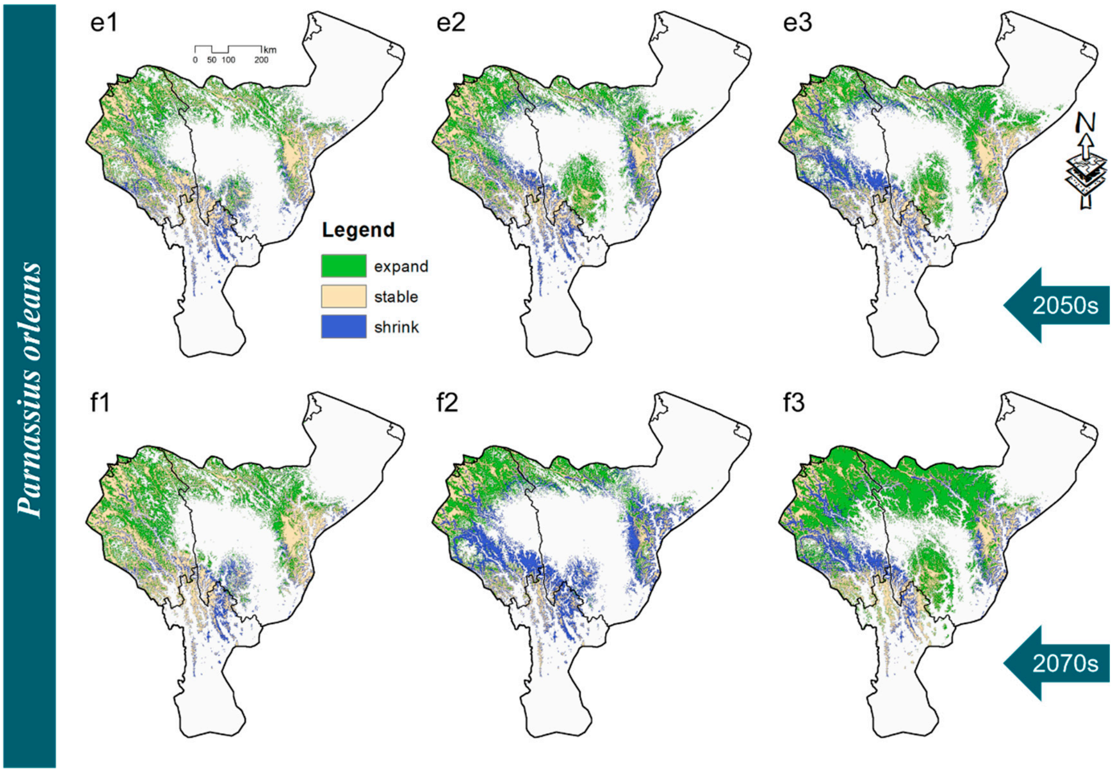

Figure S4. The projected distributional maps for the *Parnassius labeyriei*, *P. imperator*, and *P. orleans* species in the HDMs under climate change. Colors on the map show contraction (blue), stable (yellow), and expansion (red) from the current to the 2050s and 2070s, respectively, under three climate scenarios RCP 2.6 (1), RCP 4.5 (2), and RCP8.5 (3).
