# Supplementary material for: Species Richness of Papilionidae Butterflies (Lepidoptera: Papilionoidea) in the Hengduan Mountains and Its Future Shifts under Climate Change
Source: Insects. 2023 Mar 6;14(3):259. doi: 10.3390/insects14030259 (PMC10058169; doi:10.3390/insects14030259)
Supplement: Supplementary file 1 [file insects-14-00259-s001.zip › Table S1.pdf]

Table S1. Species records, AUC values under all climate scenarios, and the importance of BioClim variables in Maxent analysis, with the greatest values in red and the smallest values in green.

Species	No. of points	AUC values						
		Current	2050s			2070s		
			RCP2.6	RCP4.5	RCP8.5	RCP2.6	RCP4.5	RCP8.5
**Parnassiinae**								
Parnassius acco	36	0.902	0.904	0.893	0.901	0.918	0.916	0.927
Parnassius acdestis	26	0.904	0.922	0.917	0.904	0.899	0.903	0.884
Parnassius cephalus	90	0.856	0.881	0.855	0.855	0.879	0.876	0.888
Parnassius epaphus	44	0.791	0.821	0.826	0.788	0.796	0.764	0.785
Parnassius hide	11	0.924	0.932	0.938	0.928	0.923	0.924	0.922
Parnassius imperator	65	0.783	0.807	0.803	0.770	0.801	0.811	0.792
Parnassius labeyriei	14	0.982	0.973	0.981	0.980	0.982	0.981	0.981
Parnassius mercurius	35	0.868	0.873	0.864	0.884	0.856	0.852	0.887
Parnassius orleans	63	0.792	0.838	0.815	0.795	0.814	0.840	0.808
Parnassius simo	26	0.863	0.894	0.882	0.854	0.889	0.899	0.857
Parnassius szechenyi	28	0.865	0.864	0.867	0.864	0.879	0.888	0.885
Bhutanitis lidderdalii	19	0.982	0.982	0.982	0.981	0.982	0.982	0.980
Bhutanitis mansfieldi	9	0.962	0.939	0.969	0.950	0.970	0.973	0.949
Bhutanitis thaidina	39	0.958	0.956	0.961	0.961	0.953	0.956	0.956
**Papilioninae**								
Lamproptera curius	22	0.984	0.984	0.981	0.982	0.985	0.985	0.984
Lamproptera meges	18	0.983	0.982	0.984	0.983	0.988	0.988	0.988
Iphiclides podalirinus	13	0.976	0.975	0.975	0.980	0.958	0.958	0.967
Graphium chironides	19	0.986	0.988	0.986	0.986	0.989	0.988	0.988
Graphium cloanthus	52	0.976	0.976	0.974	0.975	0.973	0.972	0.974
Graphium confucius	9	0.853	0.858	0.854	0.840	0.927	0.926	0.919
Graphium doson	15	0.984	0.984	0.985	0.985	0.990	0.990	0.990
Graphium eurous	21	0.984	0.987	0.988	0.989	0.985	0.989	0.987
Graphium mandarinus	34	0.978	0.981	0.981	0.980	0.964	0.977	0.977
Graphium parus	19	0.983	0.986	0.988	0.983	0.977	0.987	0.984
Graphium sarpedon	40	0.980	0.985	0.980	0.979	0.974	0.973	0.974
Graphium sichuanica	8	0.987	0.988	0.988	0.988	0.989	0.989	0.989
Graphium tamerlanus	16	0.992	0.991	0.992	0.991	0.996	0.996	0.996
Teinopalpus imperialis	8	0.976	0.976	0.984	0.982	0.978	0.989	0.984
Pachliopta aristolochiae	33	0.989	0.988	0.989	0.990	0.988	0.988	0.987
Troides aeacus	42	0.963	0.960	0.962	0.962	0.961	0.962	0.964
Byasa confusus	9	0.987	0.986	0.987	0.987	0.987	0.987	0.986
Byasa daemonius	26	0.978	0.979	0.972	0.976	0.974	0.972	0.971
Byasa dasarada	26	0.985	0.987	0.983	0.984	0.991	0.990	0.991
Byasa genestieri	11	0.989	0.989	0.989	0.989	0.989	0.988	0.987
Byasa hedistus	46	0.958	0.945	0.961	0.961	0.960	0.962	0.964
Byasa latreillei	15	0.992	0.992	0.991	0.991	0.993	0.994	0.992
Byasa nevilli	83	0.975	0.976	0.974	0.975	0.978	0.979	0.980
Byasa plutonius	71	0.968	0.966	0.966	0.968	0.967	0.970	0.971
Byasa polyeuctes	101	0.959	0.957	0.962	0.960	0.961	0.962	0.962
Byasa rhadinus	12	0.989	0.988	0.981	0.983	0.986	0.985	0.982
Meandrusa lachinus	13	0.980	0.976	0.978	0.978	0.981	0.979	0.988
Meandrusa sciron	13	0.992	0.993	0.993	0.992	0.994	0.994	0.994
Papilio agestor	18	0.981	0.986	0.980	0.982	0.982	0.982	0.981
Papilio alcmenor	25	0.987	0.975	0.977	0.978	0.977	0.976	0.974
Papilio arcturus	23	0.984	0.982	0.980	0.981	0.981	0.981	0.981
Papilio bianor	60	0.972	0.968	0.972	0.972	0.970	0.969	0.969
Papilio bootes	32	0.969	0.966	0.968	0.968	0.980	0.979	0.978
Papilio epycides	37	0.978	0.979	0.978	0.980	0.977	0.979	0.981
Papilio helenus	33	0.966	0.967	0.966	0.967	0.964	0.965	0.966
Papilio krishna	18	0.965	0.961	0.966	0.964	0.983	0.983	0.983
Papilio maackii	42	0.955	0.944	0.956	0.955	0.961	0.963	0.962
Papilio machaon	56	0.952	0.945	0.954	0.952	0.950	0.957	0.955
Papilio macilentus	7	0.987	0.988	0.988	0.988	0.987	0.987	0.988
Papilio memnon	27	0.986	0.987	0.984	0.986	0.986	0.987	0.985
Papilio nephelus	22	0.978	0.979	0.977	0.977	0.980	0.979	0.979
Papilio paris	24	0.979	0.979	0.976	0.976	0.977	0.978	0.977
Papilio polytes	78	0.961	0.954	0.966	0.963	0.955	0.960	0.958
Papilio protenor	59	0.969	0.971	0.971	0.972	0.967	0.969	0.969

Papilio xuthus	77	0.963	0.960	0.966	0.963	0.958	0.960	0.959
Minimum	—	0.783	0.807	0.803	0.770	0.796	0.764	0.785
Maximum	—	0.992	0.993	0.993	0.992	0.996	0.996	0.996
Mean	—	0.954	0.956	0.956	0.954	0.957	0.958	0.957
SD	—	0.053	0.046	0.048	0.054	0.047	0.048	0.049
>0.9 (%)	—	86.4	86.4	84.7	86.4	86.4	88.1	86.4
0.8–0.9 (%)	—	8.5	13.6	15.3	8.5	11.9	10.2	10.2
