# Supplementary material for: Species Richness of Papilionidae Butterflies (Lepidoptera: Papilionoidea) in the Hengduan Mountains and Its Future Shifts under Climate Change
Source: Insects. 2023 Mar 6;14(3):259. doi: 10.3390/insects14030259 (PMC10058169; doi:10.3390/insects14030259)
Supplement: Supplementary file 1 [file insects-14-00259-s001.zip › Table S2.pdf]

Table S2. Environmental factors and their interpretation.

Code	Environment factors
Bio1	Annual mean temperature/℃
Bio2	Mean diurnal temperature range (mean (period max-min)/℃)
Bio3	Isothermality (Bio2/Bio7×100)
Bio4	Temperature seasonality
Bio5	Max temperature of warmest month/℃
Bio6	Min temperature of coldest month/℃
Bio7	Temperature annual range (Bio5-Bio6)/℃
Bio8	Mean temperature of wettest quarter/℃
Bio9	Mean temperature of driest quarter/℃
Bio10	Mean temperature of warmest quarter/℃
Bio11	Mean temperature of coldest quarter/℃
Bio12	Annual precipitation/mm
Bio13	Precipitation of wettest month/mm
Bio14	Precipitation of driest month/mm
Bio15	Coefficient of variation of precipitation seasonality
Bio16	Precipitation of wettest quarter/mm
Bio17	Precipitation of driest quarter/mm
Bio18	Precipitation of warmest quarter/mm
Bio19	Precipitation of coldest quarter/mm
