# Supplementary material for: Species Richness of Papilionidae Butterflies (Lepidoptera: Papilionoidea) in the Hengduan Mountains and Its Future Shifts under Climate Change
Source: Insects. 2023 Mar 6;14(3):259. doi: 10.3390/insects14030259 (PMC10058169; doi:10.3390/insects14030259)
Supplement: Supplementary file 1 [file insects-14-00259-s001.zip › Table S3.pdf]

Table S3. Suitable area and changes of Papilionidae butterflies in the Hengduan Mountains (HDMs) under different climatic scenarios.

Species	Suitable area (km^2)<br>Percentage of suitable area change (%)						
	Current	2050s RCP2.6	2050s RCP4.5	2050s RCP8.5	2070s RCP2.6	2070s RCP4.5	2070s RCP8.5
Parnassius acco	47334	27248<br>−42.43	20109<br>−57.52	16624<br>−64.88	23880<br>−49.55	19137<br>−59.57	8436<br>−82.18
Parnassius acdestis	65093	77713<br>19.39	18869<br>−71.01	30334<br>−53.40	75134<br>15.43	55070<br>−15.40	48081<br>−26.13
Parnassius cephalus	42652	33424<br>−21.64	36954<br>−13.36	42597<br>−0.13	39893<br>−6.47	43208<br>1.30	24642<br>−42.23
Parnassius epaphus	111058	85787<br>−22.75	58183<br>−47.61	51481<br>−53.64	103943<br>−6.41	56404<br>−49.21	47042<br>−57.64
Parnassius hide	37221	15841<br>−57.44	13267<br>−64.36	382<br>−98.97	12456<br>−66.54	0<br>−100	0<br>−100
Parnassius imperator	75971	94339<br>24.18	120058<br>58.03	182304<br>139.97	153614<br>102.20	93394<br>22.93	204969<br>169.80
Parnassius labeyriei	8984	753<br>−91.62	6<br>−99.93	0<br>−100	985<br>−89.04	1<br>−99.99	0<br>−100
Parnassius mercurius	84203	77259<br>−8.25	70079<br>−16.77	840<br>−99.00	48119<br>−42.85	36517<br>−56.63	33545<br>−60.16
Parnassius orleans	95108	123826<br>30.20	127679<br>34.25	121512<br>27.76	143347<br>50.72	86493<br>−9.06	173990<br>82.94
Parnassius simo	60759	47189<br>−22.33	22505<br>−62.96	17250<br>−71.61	45495<br>−25.12	30702<br>−49.47	7865<br>−87.06
Parnassius szechenyi	64004	26977<br>−57.85	12656<br>−80.23	7495<br>−88.29	30295<br>−52.67	20154<br>−68.51	4451<br>−93.05
Bhutanitis lidderdalii	13174	278<br>−97.89	2742<br>−79.19	7973<br>−39.48	11878<br>−9.84	19343<br>46.83	7313<br>−44.49
Bhutanitis mansfieldi	28761	50415<br>75.29	41559<br>44.50	25011<br>−13.04	36983<br>28.59	40486<br>40.77	19223<br>−33.16
Bhutanitis thaidina	17778	33641<br>89.23	18351<br>3.22	3643<br>−79.51	18333<br>3.12	10136<br>−42.99	4134<br>−76.75
Lamproptera curius	4594	10820<br>135.52	8102<br>76.36	6474<br>40.92	8392<br>82.67	4098<br>−10.80	5407<br>17.70
Lamproptera meges	2773	3491<br>25.89	2568<br>−7.39	35<br>−98.74	8094<br>191.89	10870<br>291.99	17910<br>545.87
Iphiclides podalirinus	7461	19381<br>159.76	25006<br>235.16	29360<br>293.51	15849<br>112.42	23301<br>212.30	54719<br>633.40
Graphium chironides	4259	8917<br>109.37	3793<br>−10.94	4450<br>4.48	7648<br>79.57	5676<br>33.27	2616<br>−38.58
Graphium cloanthus	11340	37970<br>234.83	18780<br>65.61	7550<br>−33.42	21883<br>92.97	18483<br>62.99	34124<br>200.92
Graphium confucius	64906	73636<br>13.45	88604<br>36.51	93053<br>43.37	69632<br>7.28	92890<br>43.11	92961<br>43.22
Graphium doson	4753	1170<br>−75.38	1035<br>−78.22	5752<br>21.02	8075<br>69.89	12735<br>167.94	5848<br>23.04

Species	Suitable area (km^2)<br>Percentage of suitable area change (%)						
	Current	2050s<br>RCP2.6	2050s<br>RCP4.5	2050s<br>RCP8.5	2070s<br>RCP2.6	2070s<br>RCP4.5	2070s<br>RCP8.5
Graphium eurous	5763	36584<br>534.81	15943<br>176.64	16001<br>177.65	8899<br>54.42	10953<br>90.06	22602<br>292.19
Graphium mandarinus	7664	27996<br>265.29	8750<br>14.17	2295<br>−70.05	24729<br>222.66	32699<br>326.66	42338<br>452.43
Graphium parus	6960	13552<br>94.71	20055<br>188.15	28303<br>306.65	12036<br>72.93	15798<br>126.98	36686<br>427.10
Graphium sarpedon	8504	15134<br>77.96	10119<br>18.99	13298<br>56.37	12794<br>50.45	8771<br>3.14	9586<br>12.72
Graphium sichuanica	4547	4903<br>7.83	4442<br>−2.31	8056<br>77.17	4737<br>4.18	3337<br>−26.61	5924<br>30.28
Graphium tamerlanus	2888	3439<br>19.08	3253<br>12.64	5687<br>96.92	3151<br>9.11	2272<br>−21.33	4468<br>54.71
Teinopalpus imperialis	4004	6099<br>52.32	9047<br>125.95	18368<br>358.74	8927<br>122.95	27426<br>584.97	46051<br>1050.12
Pachliopta aristolochiae	4618	31722<br>586.92	8465<br>83.30	0<br>−100	5468<br>18.41	1494<br>−67.65	2287<br>−50.48
Troides aeacus	8195	8350<br>1.89	5515<br>−32.70	9809<br>19.69	13139<br>60.33	14397<br>75.68	5848<br>−28.64
Byasa confusus	6007	6184<br>2.95	6220<br>3.55	7885<br>31.26	6304<br>4.94	5530<br>−7.94	6643<br>10.59
Byasa daemonius	14670	47674<br>224.98	43513<br>196.61	34298<br>133.80	28137<br>91.80	38122<br>159.86	62277<br>324.52
Byasa dasarada	6261	11604<br>85.34	6527<br>4.25	288<br>−95.40	9207<br>47.05	4082<br>−34.80	480<br>−92.33
Byasa genestieri	3245	4732<br>45.82	7625<br>134.98	2428<br>−25.18	7580<br>133.59	15932<br>390.97	37260<br>1048.23
Byasa hedistus	15092	51069<br>238.38	56189<br>272.31	12516<br>−17.07	35090<br>132.51	38169<br>152.91	56325<br>273.21
Byasa latreillei	1172	879<br>−25	103<br>−91.21	181<br>−84.56	2453<br>109.30	2083<br>77.73	731<br>−37.63
Byasa nevilli	8901	36806<br>313.50	25675<br>188.45	17005<br>91.05	20815<br>133.85	19094<br>114.52	34042<br>282.45
Byasa plutonius	11916	40885<br>243.11	30186<br>153.32	19920<br>67.17	23314<br>95.65	30333<br>154.56	35927<br>201.50
Byasa polyeuctes	13201	50927<br>285.78	34251<br>159.46	19445<br>47.30	31355<br>137.52	36898<br>179.51	46598<br>252.99
Byasa rhadinus	6242	0−100	7830<br>25.44	1709<br>−72.62	3<br>−99.95	11418<br>82.92	8467<br>35.65
Meandrusa lachinus	6576	0<br>−100	0<br>−100	3003<br>−54.33	6583<br>0.11	25391<br>286.12	433<br>−93.42
Meandrusa sciron	3802	4722<br>24.20	4749<br>24.91	7923<br>108.39	4369<br>14.91	3622<br>−4.73	6379<br>67.78
Papilio agestor	5288	9240<br>74.74	5540<br>4.77	0<br>−100	9506<br>79.77	9919<br>87.58	19265<br>264.32

Species	Suitable area (km^2)<br>Percentage of suitable area change (%)						
	Current	2050s RCP2.6	2050s RCP4.5	2050s RCP8.5	2070s RCP2.6	2070s RCP4.5	2070s RCP8.5
Papilio alcmenor	8024	22927<br>185.73	10373<br>29.27	14183<br>76.76	13482<br>68.02	10700<br>33.35	13771<br>71.62
Papilio arcturus	7252	21042<br>190.15	9283<br>28.01	11058<br>52.48	12155<br>67.61	9301<br>28.25	8702<br>19.99
Papilio bianor	9806	48932<br>399.00	40332<br>311.30	27730<br>182.79	27318<br>178.58	30883<br>214.94	52800<br>438.45
Papilio bootes	8842	31154<br>252.34	12427<br>40.55	10593<br>19.80	14962<br>69.22	8713<br>−1.46	9467<br>7.07
Papilio epycides	8733	38027<br>335.44	25057<br>186.92	1602<br>−81.66	15343<br>75.69	10066<br>15.26	30793<br>252.61
Papilio helenus	9042	22518<br>149.04	15339<br>69.64	22608<br>150.03	17143<br>89.59	17472<br>93.23	36255<br>300.96
Papilio krishna	9281	27290<br>194.04	23475<br>152.94	30681<br>230.58	19343<br>108.42	20614<br>122.11	51754<br>457.63
Papilio maackii	15659	53042<br>238.73	43995<br>180.96	44910<br>186.80	33249<br>112.33	46626<br>197.76	58662<br>274.62
Papilio machaon	15561	57868<br>271.88	44036<br>182.99	34381<br>120.94	33838<br>117.45	39679<br>154.99	73916<br>375.01
Papilio macilentus	6244	6366<br>1.95	6111<br>−2.13	10200<br>63.36	6148<br>−1.54	4520<br>−27.61	7577<br>21.35
Papilio memnon	4528	11955<br>164.02	4899<br>8.19	7385<br>63.10	8540<br>88.60	6193<br>36.77	4818<br>6.40
Papilio nephelus	6043	10834<br>79.28	5125<br>−15.19	7270<br>20.30	9749<br>61.33	7901<br>30.75	3811<br>−36.94
Papilio paris	5653	13436<br>137.68	8450<br>49.48	12777<br>126.02	11888<br>110.30	13839<br>144.81	17269<br>205.48
Papilio polytes	13325	48352<br>262.87	31844<br>138.98	16528<br>24.04	33070<br>148.18	36782<br>176.04	56759<br>325.96
Papilio protenor	8305	29844<br>259.35	17109<br>106.01	17276<br>108.02	19145<br>130.52	18670<br>124.80	29554<br>255.86
Papilio xuthus	14358	49910<br>247.61	33499<br>133.31	17508<br>21.94	36172<br>151.93	39124<br>172.49	59355<br>313.39
