# Supplementary figures and images for: Species Richness of Papilionidae Butterflies (Lepidoptera: Papilionoidea) in the Hengduan Mountains and Its Future Shifts under Climate Change
Source: Insects. 2023 Mar 6;14(3):259. doi: 10.3390/insects14030259 (PMC10058169; doi:10.3390/insects14030259)

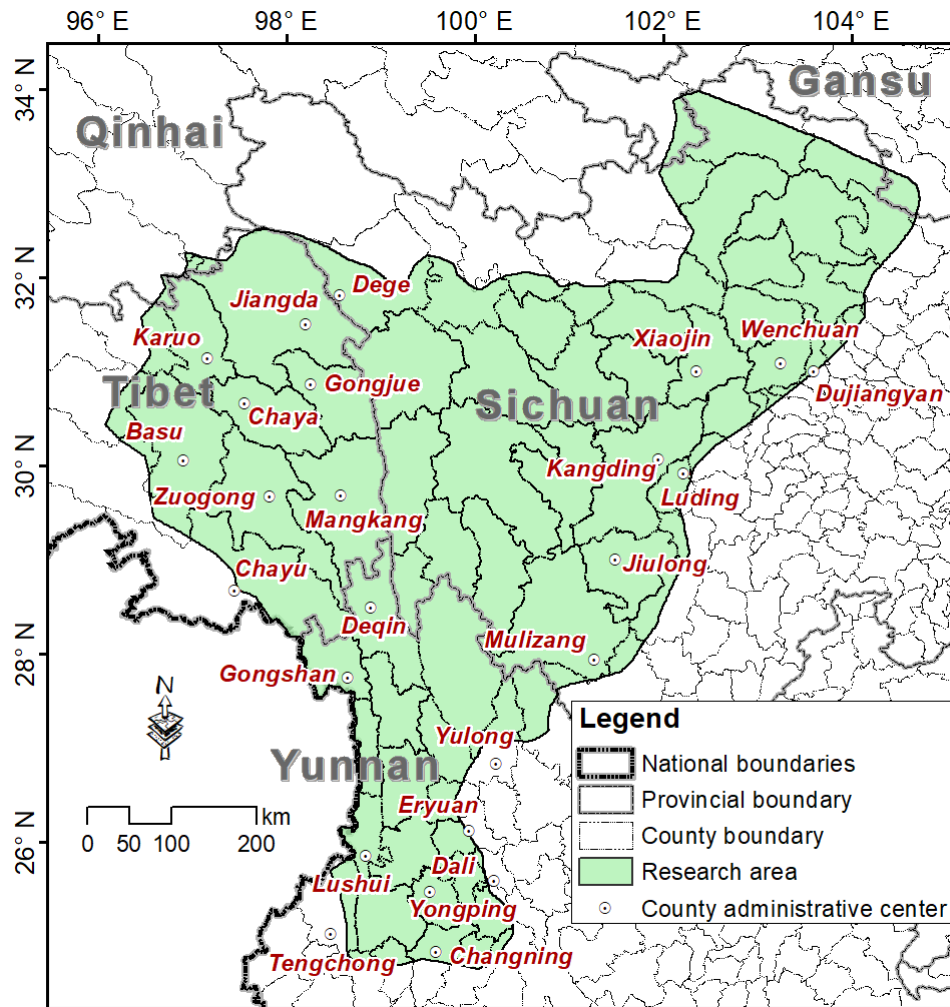

**Figure S1.** Location of the counties in the HDMs mentioned in this research

Supplement: Supplementary file 1 [file insects-14-00259-s001.zip › Figure S1.pdf]

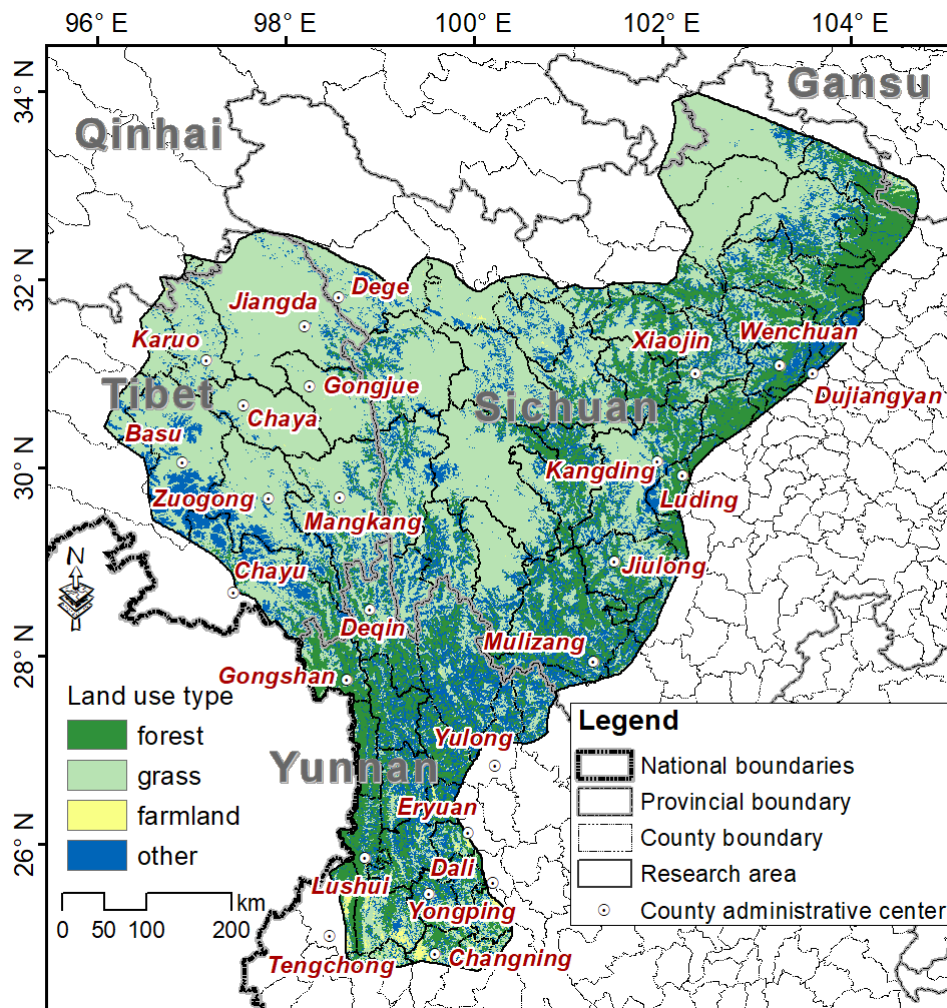

**Figure S2.** Land use types in the HDMs.

Supplement: Supplementary file 1 [file insects-14-00259-s001.zip › Figure S2.pdf]
